# Diseases of Respiratory Organs

**Published:** 1905-11-04

**Authors:** 


### PROGRESS IN MEDICINE AND SURGERY.

### DISEASES OF RESPIRATORY ORGANS.

*(Continued from page 69.)*

**Pleurisy.**—Norman Moore [4] gave a most interesting address on the pathology and diagnosis of pleurisy, quoting many interesting cases in ancient and modern times, for the diagnosis was found to be possible long before Laennec taught the value of auscultation. Still there were difficulties, and on one occasion the result of such difficulties was the death of the heir to the English Crown; for Frederick, the son of George II., developed an empyema which, being unsuspected, burst in the lung and suffocated him. Pleurisy, as Norman Moore points out, is not a mere local affection, but is generally, except in traumatic cases, an indication of some widespread morbid state. In ordinary pneumonia, tuberculosis, broncho-pneumonia, infarct, or pulmonary new growths, the nutrition of the serous membranes is so interfered with that organisms which have entered the lung are able to effect a settlement in the pleura instead of being expelled. Among the rarer causes are foreign bodies, such as nails which have slipped down the trachea and ulcerated through; and the bacilli of plague and diphtheria. Tuberculosis, indeed, sometimes enters from the abdomen through the diaphragm, though the common route through the lung is of clinical importance, since a slight pleurisy is often one of the early manifestations of phthisis while it is in a curable stage. The diagnosis of pleurisy often presents some difficulty; thus we may mistake the pain of herpes zoster or of cardiac disease for pleurisy. Lobar pneumonia may be mistaken for it, and though the rigors, the pyrexia, and the onset of a pneumonia are generally more severe, owing probably to the greater intensity of the infection, yet bronchial breathing itself may be present in a pleurisy if solid exudate is plentiful and little fluid is present. Large serous effusions are often the result of either tubercle or the pneumococcus, while an empyema may follow a tuberculous affection or a lobar pneumonia, and in the case of children a lobar pneumonia may show no definite physical signs whatever. A curious symptom has been noted by French writers in the inequality of the pupils. This is not constant, but when present the dilated pupil is generally on the side of the effusion. Chauffard and Loederich [5] think it is due to the irritation of the branches of the vagus distributed to the lung on the affected side. They found it in seven out of seventeen cases, but it seems to have no relation to the amount of effusion or to the nature of the pleurisy.

**Treatment of Effusion.**—S. West [6] points out that absorption into the blood-vessels takes very little part in the removal of serum. The pleura is a lymph sac with valves directed away from the cavity, and the fluid which exudes from the blood-vessels is in health constantly being pumped out into the lymph trunks by the movements of respiration. When great effusion has taken place it not only presses upon the superficial lymphatics, but hinders the movements of the thorax until the pump ceases work altogether. Hence the curious disappearance of the fluid which sometimes follows the removal of a very small portion, though in practice the more we take away the better. It has been said that early tapping shortens the duration of an attack, but West points out that the febrile condition runs its course, whether paracentesis has been done or not. It might be said, in reply, that though an immediate fall does not follow the operation, we need to compare the duration of many cases which had been left alone with that of a similar number which had been tapped, to say whether the duration has been really shortened. Dyspnœa may arise from (1) a rapid increase of fluid before the lung has time to accommodate itself, or, what is more important (2) from functional congestion and breakdown of the opposite lung. In either case it is an indication for immediate tapping. West holds that indications against tapping do not exist, and that it is best performed by a tube reaching down to the floor instead of by an aspirator, since there is less violence employed on the lung, less danger of pneumothorax or of shock and cardiac disturbance.

**The Treatment of an Empyema.**—West's rules are interesting to compare with those of some surgeons. He lays down that a free incision should be made as early as possible, though occasionally in a risky case paracentesis will gain time for the patient to be brought into a better state, and, indeed, paracentesis alone does sometimes result in a complete cure. Here, of course, an aspirator must be used. In an operation a needle must always be passed first and the pus found by it. The incision is preferably made at the level of the nipple just in front of the postaxillary border. Resection of a rib should not be done if drainage can be obtained without it. Washing out the pleura is without danger and should be done whenever the pus is curdy, flaky, offensive, or contains blood clot. The tube should not be taken out too soon, but the cavity should be examined with a probe every few days. The track should close up from the bottom leaving no cavity behind it. The physician himself should attend to the management of the case. The results of Estlander's operation are most unsatisfactory.

**In Pneumothorax** tapping may be needed immediately to give exit to the air and relieve the dyspnœa. Syphonage should be alone employed and repeated if necessary. If this fails, free incision may be required. Secondly, if air and fluid are present later on, paracentesis by the syphon may be needed as in simple pleurisy. Here care must be used that the mouth of the syphon be kept below the surface of the water in the bowl, otherwise the air alone and not the fluid will be drawn out. Pyopneumothorax should be treated under just the same laws as an empyema—namely, early and free incision whether tubercular or not.

**Interlobar Empyema.**—The difficulty of diagnosis is great and probably many cases are overlooked. Occasionally the pus makes its way outwards into the general cavity or arrives at the surface of the body. Sir W. Broadbent [7] suggests that cases where the pus has discharged into a bronchus without pneumothorax are probably due to such a collection between the lobes. Occasionally shifting dullness with the absence of fluid in the lower part of the chest may point to such an empyema in the presence of general symptoms. Puncture high up in the axilla has been successful in reaching a collection in two cases after several unsuccessful attempts had been made.

[4] Lancet, June 10. [5] Ibid., April 6. [6] Ibid., March 25. [7] Pract., February.

